# Albinism in barley androgenesis

**DOI:** 10.1007/s00299-013-1543-x

**Published:** 2013-12-11

**Authors:** Katarzyna Makowska, Sylwia Oleszczuk

**Affiliations:** Plant Breeding and Acclimatization Institute, National Research Institute, Radzikow, 05-870 Blonie, Poland

**Keywords:** Androgenesis, Barley, Plastids, Albinism, Microspore, Albino plants

## Abstract

Androgenesis is highly useful for plant breeding, significantly reducing breeding cycle times, as well as in a wide range of biological research. However, for widespread use this process must be efficient. Despite several decades of research on the phenomenon of androgenesis, many processes involved are obscure and there is much to be understood about androgenesis. One of the problems inherent in androgenesis, and reducing its efficiency, is albinism. This article reviews albinism in barley anthers and microspores in vitro cultures. Of special interest is the fate of plastids throughout androgenesis, which is important at several levels, including the genes responsible for driving the green-to-albino ratios. We also summarize the external factors that reduce the incidence of albino plants that are regenerated via androgenesis.

## Introduction

In vitro androgenesis in plants was first performed almost 50 years ago (Guha and Maheshvari 1964). This process involves the development of androgenic embryos from cells of male gametophytic pathway––the microspores. Microspores at an appropriate developmental stage subjected to different types of exogenous stresses can be reprogrammed. The developmental pathway change from gametophyte to sporophyte results in the formation of androgenic embryos (Jähne and Lörz 1995; Touraev et al. 2001). After chromosome doubling these plants become doubled haploid (DH), which means that they can reasonably be expected to be homozygous at every genetic locus. DH plants are an attractive material for a variety of research and breeding programs including the development of new cultivars, genetic mapping, localisation of quantitative traits loci (QTL) (Devaux and Pickering 2005), transformation (Kumlehn et al. 2006) and embryological studies (Wróbel et al. 2011). Additional applications include the development of specific chromosome stocks, specifically the cases where gametic selection reduces male transmission of specific chromosomes (for example, the development of disomic alien addition lines).

Owing to a large number of microspores present in a single anther, and a large number of anthers per spike, the expectation appears reasonable that the number of androgenic plants generated from a single spike would be in the tens of thousands. However, in a plant such as barley, the number of regenerated green plants per 100 responding anthers can range from zero to several hundred, depending on the genotype (Jacquard et al. 2009; Li and Devaux 2003). The reasons for such a disparity are due to a variety of factors, including the ability of microspores to alter their developmental pathway, the survival rates of in vitro cultures, the efficiency of androgenic embryo production, and the regeneration ability to form green plants, as well as the highly important phenomenon of albinism.

Albinism is defined as a plant’s inability to produce chloroplasts and thus carry out the process of photosynthesis. Chlorophyll-deficient plants are able to achieve only the early stages of development when grown on artificial media under the in vitro culture conditions. Albinism often occurs in androgenesis-derived plants, including most cereals, such as wheat (Liu et al. 2002), rye (Immonen and Anttila 2000), triticale (Oleszczuk et al. 2004), oat (Kiviharju et al. 2000) and barley (Caredda et al. 2004). Within the same species, there are often cultivars that are more susceptible to albinism than others. In the most albinism-prone cultivars, all regenerated plants lack chloroplasts, whereas in other cultivars, the fraction of albino plants is insignificant (Li and Devaux 2003). In barley, the fraction of albino plants ranges from 1 to 99.7 % depending on the genotypes (Caredda et al. 2000; Castillo et al. 2000). Interestingly, it has been observed that in barley, albinism is more widespread in spring than in winter cultivars.

The direct cause of albinism in androgenesis-derived plants is the inability of proplastids to transform into chloroplasts; the biological mechanism has not been elucidated. Research to elucidate the mechanism of albinism has focused on three areas: cytology, plastid genomics and the nuclear genome-focused studies. Cytological studies aim to identify structural changes in plastids at different stages of androgenesis that prevent chloroplast formation (Caredda et al. 1999, 2000, 2004). Genome studies focus on the identification of differences in the genomes of green and albino plant plastids (Hofinger et al. 2000). Nuclear genes, which are responsible for a large number of albinos in some barley cultivars, have also been identified as culprits in some studies (Muñoz-Amatriain et al. 2008, 2009). The distinction into three areas of study is arbitrary, created for the purpose of this review, as in real life there are likely interactions among the three areas.

## Cytological studies on albinism

The research of Caredda and co-workers (1999, 2000, 2004) made a significant contribution to the understanding of plastid physiology in microspores prior to the switch to sporophytic development, at various times during androgenesis itself and in the regenerated plants. Their research focused on two barley cultivars, winter cv. ‘Igri’ and spring cv. ‘Cork’: both show similar numerical parameters of androgenesis but differ significantly in terms of the green to albino ratio among regenerated plants. The ratio for Igri was 7.2 and 0.03 for Cork, which translated to 99.7 % Cork regenerants being albinos (Caredda et al. 2000). The authors observed that even before the reprogramming stress was applied, there were structural differences between plastids in vacuolated microspores of the two cultivars. In Igri, plastids were much larger and three times as numerous as in Cork. Moreover, there were more thylakoid membranes and starch grains in Igri. The authors concluded that plastid development may be determined at the early stages of microsporogenesis. In Cork, plastids in vacuolated microspores had relatively small amounts of ptDNA and were programmed for the gametophytic development pathway that under normal physiological conditions leads to plastid elimination. Hence, these plastids could not be reprogrammed to undergo the sporophytic development. Plastids of Igri represented an earlier stage of development, making them more prone to alterations in developmental.

The critical juncture for chloroplast biogenesis in androgenesis is the reprogramming change in the microspore developmental pathway (Shariatpanahi et al. 2006). Exogenous stress causes major changes to plastid physiology. One such change is a drastic reduction in the number of plastids in microspores, and the rate of reduction depends on type of stress applied. Greater reduction was observed after cold shock than after osmotic stress (Caredda et al. 1999). Changes in plastid structure can be observed after ~12 days on the induction medium. At this time, some plastids increase in size, new starch grains and thylakoids appear which, in the following days of culture, arrange themselves into grana. The rate of divisions in these plastids increases, and they turn into chloroplasts. However, in some plastids, the internal changes are limited to accumulation of starch grains, while their network of internal membranes does not undergo expansion and the rate of division decreases. These changes are typical for plastids that do not undergo the shift to androgenic development. These plastids continue to function in the same manner as during normal formation of pollen, which eventually leads to regeneration of albino plants (Caredda et al. 2000). Therefore, the accumulation of starch grains at these very early stages does not correlate with regeneration of chlorophyll-deficient plants, perhaps due to excessive levels of metabolism of the medium carbohydrates by the microspores (Wojnarowiez et al. 2004).

According to Caredda et al. (1999), in Igri, the number of plastids during growth and development of androgenic structures on the regeneration medium was 10 times higher than that in Cork. In the latter, a spring cultivar, most plastids are transformed into amyloplasts. In these amyloplasts, the starch comprises 90 % of the total plastid volume, which is 3.5 times higher than that of Igri. The ability of Igri plastids to divide is much higher than that of Cork.

Green plants possess numerous chloroplasts with highly developed internal membrane systems and few starch granules. By contrast, plastids of albino plants have poorly developed internal membrane systems, large numbers of prolamellar bodies and plastoglobules do not contain starch grains (Caredda et al. 2000, 2004). Differences in plastid structure during development into green and albino plants are listed in Table 1. The changes in plastids during consecutive phases of androgenesis illustrate the complexity of albinism. It is apparent that stress is capable of reprogramming the microspore itself but not necessarily the plastids themselves. In this sense, it appears that the developmental programs between microspore and its plastids are independent. Reprogramming may lead to differences in the developmental fate when microspores undergo cell division, and this may lead to the production of androgenic embryos (the sporophytic pathway) containing plastids that follow the gametophytic pathway, and this leads directly to regeneration of chlorophyll-deficient plants.

**Table 1:**
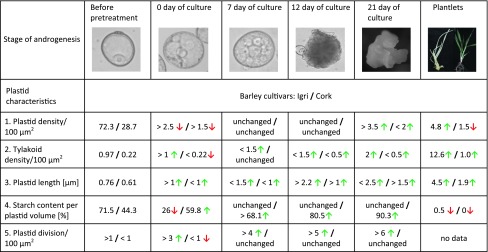
Changes in plastid structure during in androgenesis

Based on Caredda et al. (1999, 2000, 2004), with modifications. *Arrows* indicate the trend relative to the previous column

Presumably, the roots of albinism can be found at the early stages of androgenesis (Muñoz-Amatriain et al. 2009). However, Mouritzen and Holm (1994) postulate that changes in the plastid genome that lead to albinism take place only during the plant regeneration stage, at the point of differentiation and the apical meristem formation. If albinism indeed has its roots in early androgenesis, changes in the in vitro culture conditions should not significantly affect the ratio of regenerated green to albino plants. However, many reports describe these types of dependencies (Jacquard et al. 2009; Kumlehn et al. 2006). If an assumption is made that plastid development is determined at a very early stage of microsporogenesis, the only way to increase the green to albino ratio by altering culture conditions would be to increase the relative survival rate of microspores that contain plastids capable of becoming chloroplasts. To date, no observations to support this hypothesis have been reported.

## The plastid genome in androgenesis

Changes in the plastid genome and transcription levels of the plastome-coded genes may explain why it is impossible to convert various plastids into chloroplasts. Studies of this phenomenon are based on the comparison of the plastid genome structure between green and albino plants produced via anther or microspore culture. However, the volume of scientific data on the androgenesis-triggered plastome rearrangements in barley is limited and so it makes sense to examine relevant studies from other plant species.

Plastid genomes of green plants are considered highly stable. By contrast, among chlorophyll-deficient plants regenerated by androgenesis, large defects in the ptDNA structure are often detected. These defects are predominantly single or multiple deletions of DNA segments, ranging from 10 to 100 kbp (Harada et al. 1992). The size of deletions may be dependent on the regeneration method, with more alterations in the callus-derived plants than in those obtained directly from androgenic embryos (Day and Ellis 1985). Most deletions occur in the adenine- and thymine-rich regions, mainly in the large single copy region, which is the least stable region in the entire plastome genome. This region primarily contains genes responsible for photosynthesis, e.g., genes encoding the proteins of photosystem I and II. The deletions may also alter the conformation of the remaining ptDNA that may lead to additional changes in transcription (Dunford and Walden 1991; Hofinger et al. 2000). Not all segments of ptDNA are subject to deletions. This is particularly true for segments situated in the inverted repeat regions (IR), which are primarily associated with DNA replication (Dunford and Walden 1991) or heme synthesis (Harada et al. 1992). Interestingly, these regions are conserved among albino plants of a given line or cultivar as well as among cultivars of other species such as rice, wheat and barley (Day and Ellis 1984, 1985).

The exact mechanisms by which these rearrangements occur during androgenesis are largely unknown but it has been proposed that the presence of truncated *ycf1* gene sequences can play a role in triggering the plastid genome rearrangements in monocots (Ankele et al. 2005). Nevertheless, some researchers have reported that changes in ptDNA are not necessarily present in all albino plant plastids. Indeed, ptDNAs with restriction patterns identical to those of green plants have been identified (Dunford and Walden 1991; Hofinger et al. 2000), implying the absence of deletions or other major rearrangements.

Taking into account the variable nature and sizes of plastome rearrangements one could expect that changes in the plastid gene expression patterns reflect the deleted plastome fragments. On the contrary, the expression patterns appear to be similar in albino plants with assorted deletions or even those with apparently intact plastome. The common features of albino plants’ gene expression patterns include low transcription levels of the photosynthesis-related genes, including Rubisco, and upregulated transcription of the housekeeping genes. It has been noted that often even when the IR regions of the plastome are preserved, the transcription levels of of the IR-coded ribosomal RNA is always low (Ankele et al. 2005; Hofinger et al. 2000).

Taken together, these observations suggest that deletions in ptDNA may be not the primary cause of albinism. Instead, they may only represent a reaction to some stressor, such as the microspore reprogramming stress or stresses related to the in vitro culture itself (Touraev et al. 2001). Hofinger and co-workers (2000) proposed that the altered transcription levels were the event preceding plastome rearrangements with a reduced activity of the plastome-coded RNA polymerase (PEP) playing a key role. Since studies of ptDNA rearrangements have primarily focused on regenerated plants, a key question remains unanswered: when (how early in androgenesis) does the ptDNA disorder occur?

Apart from the rearrangements of the plastome, changes were also identified in the transcription levels of the nucleus-coded chloroplast-localised proteins (Dunford and Walden 1991). This points to the important role of the nuclear genome in abnormal plastid function in the androgenesis-derived albinos.

## The effect of the nuclear genome on albino generation

Differences in the level of albino plant regeneration among barley genotypes indicate that the tendency to form chlorophyll-deficient plants is, to a degree, genetically determined. Based on crosses between albinism-susceptible and albinism-resistant cultivars, it has been concluded that the genes responsible for this character are inherited in a Mendelian fashion and hence, they must be nuclear (Larsen et al. 1991). Many studies have focused on the identification of genetic loci associated with the ratio of green to albino regenerants obtained via androgenesis. Identification of quantitative trait loci (QTLs) associated with albinism, and DNA markers linked to these QTL may enable rapid assessment of suitability of any specific cultivar for androgenesis. Perhaps, in the future, a simple genetic screen will help to identify material especially susceptible to albinism without resorting to androgenesis itself. In barley, two QTL associated with the number of androgenic embryos and albino plant production were located, one on chromosome 3H and the other on 5H. However, only one of these has been employed to predict the percentage of green plants in 30 commercial cultivars, but it explained only 20 % of the variation between genotypes (Muñoz-Amatriain et al. 2008). In a different set of mapping populations of barley, more QTL have been identified: on chromosome 2H and associated with the number of embryos and albino plants and on chromosomes 5H and 6H and correlated with regeneration of green plants (Chen et al. 2007). The approach of looking for specific genome regions (QTL) can be effective, but accurate parental genome-based predictions of chances for high proportions of albinos require additional QTL that explain high-levels of variation. The obvious major problem with this approach is that it is very uncertain how many genes affect the production of albino regenerants.

The effect of nuclear gene expression on albino formation during androgenesis has been examined using microarrays. In one such experiment, the gene expression profiles in barley genotypes with contrasting green to albino ratios were compared in anthers immediately after application of androgenesis-induced stress (Muñoz-Amatriain et al. 2009). The two genotypes showed significantly different expression patterns of genes affecting plastid differentiation. However, there were no differences in the expression patterns of genes involved in carbohydrate metabolism. This suggests that the accumulation of starch grains in plastids of albino plants is a late-occurring, secondary phenomenon, and that the developmental fate of plastids is determined at an early stage of androgenesis. Like most microarray experiments, this experiment also produced huge amounts of data that were difficult to interpret, also because the functions of a large portion of the transcripts analysed are unknown. Nonetheless, this study helps to confirm the hypothesis that nuclear genes affect plastid development during haploid embryogenesis. Such results would enable us to quickly determine the expected albinism level of a cultivar using genetic engineering tools and may improve our understanding of the mechanisms leading to the albino plant regeneration.

## The impact of external factors on the number of albinos

The efficiency of androgenesis is usually defined as the number of green plants obtained per starting material. The efficiency can be improved by manipulation of appropriate external factors. Only some of these factors have been examined in the context of albinism (Olmedilla 2010), with most studies focusing on selection of a proper type of the reprogramming stress and proper proportions of components in the induction medium. In barley, the most commonly used stressors include either cold treatment alone, or osmotic stress combined with low or high temperature (Oleszczuk et al. 2006). Identifying suitable pretreatment conditions is difficult, as the stress must be strong enough to alter the microspore developmental pathway, yet gentle enough not to result in high mortality or to disturb cellular function. The matter is not trivial: properly chosen reprogramming stress reduced the number of albinos by ~67.8 % in barley cv. ‘Scarlett’ (Oleszczuk et al. 2006). In some reports, osmotic stress was used, by addition of various compounds such as mannitol, sorbitol, sucrose or polyethylene glycol (PEG) to the solution in which the anthers were incubated. Wojnarowiez et al. (2004) compared the effects of these osmotic stressors on the level of albinism in barley and showed that the type of androgenesis-inducing compound employed affected the number of albino plants obtained. In Igri, the lowest percentage of albino plants was observed with sorbitol (4.9 % albinos) while mannitol, sucrose or PEG was much worse (12.2, 15.6 and 17.7 %, respectively). However, even the worst of the four treatments was a significant improvement over the control, 52 % of the regenerated plants were albinos. Moreover, plastids of plants treated with mannitol and PEG did not accumulate starch grains at any stage of androgenesis. On the other hand, sucrose and sorbitol resulted in the accumulation of significant numbers of starch granules in plastids, which did not always correlate with regeneration of albino plants.

In experiments aiming at the reduction of albinism, promising results were obtained with the addition of copper sulphate to the medium. Copper ions play an important role during pollen development, by affecting the metabolism of tapetal cells and microspores. Copper ions also take part in biosynthesis of chlorophyll, as well as in photosynthesis (Jacquard et al. 2009; Wojnarowiez et al. 2002). Appropriate concentration of copper sulphate in the ‘stressing’ and inducing media favourably affects all parameters of androgenesis, i.e., the microspore survival after activation from stress, the number of responsive anthers, plant regeneration and the ratio of green: albino plants. Moreover, it affects the division of plastids and the parallel development of internal membranes, and induces the transformation of amyloplasts into proplastids. Copper sulphate reduced the number of albino plants by 2.2 % relative to the control in Igri and by 18.7 % in Cork. Importantly, green plants were obtained from such cultivars where previously only albinos could be regenerated (Jacquard et al. 2009; Wojnarowiez et al. 2002).

The effects of carbohydrates in the media have been widely studied (Jähne et al. 1991; Scott et al. 1995). According to some authors, carbohydrates, which are a source of carbon and regulate the osmotic pressure in the culture, are the most important components of the induction medium. The types of carbohydrates used have an impact on the survival of microspores, the number of androgenic embryos produced and the ratio of green to albino plants (Cai et al. 1992). Sucrose has a negative effect on androgenesis in barley, as it affects the level of starch accumulation in microspores and its degradation products are toxic to microspores (Wojnarowiez et al. 2004). Replacing sucrose with maltose as the carbon source has a positive effect, improving the ratio of green to albino plants (Tiwari and Rahimbaev 1992).

Many attempts to reduce albinism have been undertaken, some of which were more effective than others. The addition of zinc sulphate to the ‘stressing’ and induction media increased the number of androgenic embryos but did not have much of an effect on the ratio of green to albino plants (Echavarri et al. 2008). Glutamine, as a source of organic nitrogen, has a positive effect on the regeneration of plants from microspores (Hoekstra et al. 1992). Subsequent studies have shown that the lack glutamine in the induction medium not only improves the efficiency of plant regeneration, but also significantly reduces the number of albinos (Castillo et al. 2000; Ritala et al. 2001). Studies examining the effects of stress and culture conditions on albino formation during androgenesis are summarised in Table 2. These studies demonstrate that by optimising various parameters of the in vitro culture, some reduction in the frequency of albinos is possible. However, the impact of external factors is much smaller than that of the genotype.

**Table 2:** A summary of research on the impact of stress and culture conditions on albinism in barley

Cultivars of barley	Phase of androgenesis	Range of research	Results		Reference
			% GP in control	% GP studied groups	
Cork	Pretreatment	Different concentrations of copper sulphate	1.4	0.0–20.1	Jacquard et al. (2009)
Douchka			0.0	0.0–4.1	
Igri	Pretreatment	Different concentrations of zinc sulphate	93.6	86.6–92.7	Echavarri et al. (2008)
Reinette			29.4	28.1–56.0	
Scarlett	Pretreatment	Different concentrations of mannitol, temperature and time of pretreatment	28	7.1–96	Oleszczuk et al. (2006)
Igri	Pretreatment	Different types of carbohydrate	47	52.4–87.8	Wojnarowiez et al. (2004)
Hop	Pretreatment	Different concentrations of mannitol pretreatment/cold pretreatment	10	15–33	Cistue et al. (1998)
Reinette			8	43–57	
Igri	Induction medium	Different sources of iron	35.09	0–17.58	Novotny et al. (2000)
Novum			0.47	0–1.84	
Hispanic	Induction medium	Liquid/solid induction medium	0.6	84	Li and Devaux (2003)
Lyric			55	76	

Phase of androgenesis: pretreatment indicates stress culture conditions applied to change the microspores’ development pathway from gametophytic to sporophytic; induction medium indicates culture conditions applied during the development of androgenic embryos. Range of research indicates the type of intervention or tested changes of conditions; % GP indicates the percentage of green plants among all plants obtained

## Final remarks

Albinism in haploid embryogenesis has been a hard problem to understand and solve. The crucial and still puzzling event appears to be the reprogramming of microspores from the gametophytic to sporophytic developmental pathway. It is not yet known how stress alters the developmental pathway, or which genes are responsible for this transition. Even more puzzling is how androgenesis disorders lead to albino plant formation. The behaviour of plastids during androgenesis has already been described: some plastids develop into chloroplasts; others develop into amyloplasts. The failure of not developing into chloroplasts may be a consequence of failed reprogramming. To date, no specific type of stress has been identified that would reliably reprogram plastids. At least two questions remain unanswered. (1) When a microspore is exposed to stress, must all plastids be reprogrammed to regenerate a green plant or will only some proportion do? Does the applied stress act in a binary way, i.e., by either changing the developmental pathway in all plastids or none of them? (2) If the hypothesis of Caredda and co-workers is accepted on the absence of reprogramming in plastids, why are proplastids present in the regenerated plants while amyloplasts are not found? Several studies suggest the importance of the developmental stage of the microspores at the beginning of androgenesis. Perhaps making use of microspores at the early stages of development would reduce the number of albinos produced. To date, there are no data to support this assumption.

Information on the modifications in the plastid genome comes from analyses of albino plants. It is not known when these changes take place. It would be quite interesting to observe the changes that occur in the plastome from before the pretreatment through plant regeneration. Do the plastid genome rearrangements occur immediately after the application of stress or only during the subsequent days in in vitro culture? Perhaps some rearrangements occur before microspore reprogramming. Such information could help answer many questions, including the following: what is the direct source of rearrangements in the ptDNA?

Genes that affect differences in the green to albino ratios between species and cultivars are known to be encoded by the nuclear genome. However, only few loci affecting this trait have thus far been identified, and many more such loci almost certainly exist. A complete understanding of the causes of albinism and the changes leading to the albino plant formation would make it possible to manipulate the in vitro culture conditions and perhaps eliminate this undesirable phenomenon.

Although barley androgenesis has been known and used for some 50 years, the process still remains quite mysterious. It is hoped that with rapidly developing molecular biology techniques and better laboratory equipment, many riddles of haploid embryogenesis, particularly the phenomenon of albinism, will one day be solved.

## Abbreviations

DH: Doubled haploids
ptDNA: Plastid DNA
QTL: Quantitative trait loci
PEG: Polyethylene glycol
IR: Inverted repeat regions
